# Using Tournament Angler Data to Rapidly Assess the Invasion Status of Alien Sport Fishes (*Micropterus* spp.) in Southern Africa

**DOI:** 10.1371/journal.pone.0130056

**Published:** 2015-06-05

**Authors:** John S. Hargrove, Olaf L. F. Weyl, Micheal S. Allen, Neil R. Deacon

**Affiliations:** 1 Department of Wildlife Ecology and Conservation, University of Florida, Gainesville, Florida, United States of America; 2 South African Institute for Aquatic Biodiversity (SAIAB), Grahamstown, South Africa; 3 Centre for Invasion Biology, SAIAB, Grahamstown, South Africa; 4 Fisheries and Aquatic Sciences Program, University of Florida, Gainesville, Florida, United States of America; Bournemouth University, UNITED KINGDOM

## Abstract

Fishes are one of the most commonly introduced aquatic taxa worldwide, and invasive fish species pose threats to biodiversity and ecosystem function in recipient waters. Considerable research efforts have focused on predicting the invasibility of different fish taxa; however, accurate records detailing the establishment and spread of invasive fishes are lacking for large numbers of fish around the globe. In response to these data limitations, a low-cost method of cataloging and quantifying the temporal and spatial status of fish invasions was explored. Specifically, angler catch data derived from competitive bass angling tournaments was used to document the distribution of 66 non-native populations of black bass (*Micropterus* spp.) in southern Africa. Additionally, catch data from standardized tournament events were used to assess the abundance and growth of non-native bass populations in southern Africa relative to their native distribution (southern and eastern United States). Differences in metrics of catch per unit effort (average number of fish retained per angler per day), daily bag weights (the average weight of fish retained per angler), and average fish weight were assessed using catch data from 14,890 angler days of tournament fishing (11,045 days from South Africa and Zimbabwe; 3,845 days from the United States). No significant differences were found between catch rates, average daily bag weight, or the average fish weight between countries, suggesting that bass populations in southern Africa reach comparable sizes and numbers relative to waters in their native distribution. Given the minimal cost associated with data collection (i.e. records are collected by tournament organizers), the standardized nature of the events, and consistent bias (i.e. selection for the biggest fish in a population), the use of angler catch data represents a novel approach to infer the status and distribution of invasive sport fish.

## Introduction

Organisms transferred outside their natural range that establish self-sustaining populations pose potential threats to native flora and fauna worldwide [1]. Invasive species are considered a significant driver of global biodiversity change [2–4] and for this reason monitoring invasions is a management concern for conservation authorities [5]. Several frameworks have been developed to assess the invasion status based on evidence of introduction, survival, reproduction in the wild, and spread from the point of introduction (see [6]). While specific phases (e.g. introduction) are well documented for many invasive species (e.g. Global Invasive Species Database, www.issg.org/database), there is often less information on their subsequent establishment, spread, and abundance [7]. In addition, many invasions are unintended or unauthorized, and collecting the appropriate biological information to assess invasion state is a bottleneck in the assessment process [8].

Fishes represent one of the most commonly introduced aquatic taxa worldwide [9]. In countries such as the United States with well-resourced fisheries departments, considerable resources are directed towards monitoring population abundances of species used in fisheries [10], and the distribution of fishes is generally well documented [11]. However, due to incomplete detection, monitoring methods may fail to detect changes in occurrence and abundance of invasive species [12]. In southern African countries, fish monitoring efforts are less extensive (e.g. [13]) and many fish introductions remain undocumented because they are undertaken informally, often by individuals seeking to develop opportunities for angling [14].

Information on the introduction, establishment, and spread of invasive fish species in southern Africa is an urgent requirement for understanding and managing invasions [15]. The prospect of undertaking large scale assessments in expansive geographic regions such as southern Africa represents a formidable task given the large numbers of water bodies and paucity of personnel and resources. As a result, alternative means of determining distribution and status of freshwater fishes warrant consideration. For sport fishes, such as bass (a collective term for largemouth bass *Micropterus salmoides*, smallmouth bass *Micropterus dolomieu*, spotted bass *Micropterus punctulatus* and Florida bass *Micropterus floridanus*), angler catch data may provide important insights into their distribution and establishment success. In their native range (North America), bass are the focus of a multi-billion dollar sport fish fishery [16] and catch data associated with bass angling tournaments have been used to evaluate the size structure and population density [17,18], primarily to guide fisheries management interventions [19,20]. As a result of their introduction into inland waters in Asia, Europe, South America, and Africa [21–23], professional tournaments organizations such as the Bass Anglers Sportsman Society (B.A.S.S.) now operate on a global scale (e.g. [24]).

In this paper, tournament angling data was used to assess the invasion status of black bass *Micropterus* spp. in southern Africa. First, the distribution of black bass in southern Africa was mapped using tournament event locations held in Botswana, Mozambique, Namibia, South Africa, and Zimbabwe. Secondly, the utility of tournament catch records to assess invasion status for sport fishes was evaluated. To achieve this objective, we used tournament catch records to test whether (1) catch rates, an indicator of relative abundance [25]; and (2) individual fish weights, an indication of individual fish size, differed between invasive (southern Africa) and native (U.S.) populations.

## Materials and Methods

### Mapping the distribution of black bass in southern Africa

Geographic locations were compiled for competitive angling tournaments held in Botswana, Mozambique, Namibia, South Africa, and Zimbabwe. Data were drawn from a variety of sources and included bass fishing organizations operating at the national, regional, and local level. Although this allowed for discrepancies in tournament formats followed by governing organizations (e.g. length of fishing day, number of anglers per angling team, maximum number of fish retained), the fundamental aspects of tournament angling were similar in all cases (i.e. artificial lures only, hook-and-line angling only, anglers competing to capture the heaviest limit of fish, [24]). All tournaments from which angling data was collected required participants to follow appropriate fishing regulations and possess relevant permits. Latitude and longitude data for water bodies were obtained from Google Earth and geographic data projections were generated using ArcGIS v.10.2.2 (ESRI, Redlands, CA, USA).

### Population success of alien-invasive black bass in southern Africa relative to native populations

The success of alien invasive centrarchid populations in southern Africa were evaluated relative to native populations in North America using only data from tournaments organizations directly affiliated with the Bass Anglers Sportsman Society (B.A.S.S.) in the USA because they enforce a common set of rules on fishing effort per day, number of anglers, and number and minimum size of fish brought to judging stations. Southern African B.A.S.S. affiliated organizations include the South African Bass Angling Association (S.A.B.A.A.), the Zimbabwe National Bass Federation (Z.N.B.F.), and the Namibian Bass Angling Association (N.B.A.A.). Tournament formats are identical to those in B.A.S.S. tournaments in the USA with respect to the length of tournament day (generally 7 to 10 h), tournament length (2–3 days), acceptable forms of fishing tackle (rod length, lure type, etc.), appropriate means of fish capture (artificial bait only), minimum size of fish that may be retained (generally 30 cm total length TL), maximum number of fish per angler (5 fish limit), and boating requirements (operation and safety equipment requirements). Tournament records are kept at each event, and include the numbers and weights of fish caught by each angler on a daily and overall tournament basis. Bass populations in Zimbabwe and Namibia consist of populations of pure *M*. *salmoides*, *M*. *floridanus*, and *M*. *salmoides* x *floridanus* intergrades (N. Deacon, personal observation), whereas South Africa reservoirs included in analysis have populations of pure *M*. *salmoides*, *M*. *salmoides* x *floridanus* intergrades, and two populations of *M*. *dolomieu* (Clanwilliam and Quaggaskloof dams) (O. Weyl, personal observation).

Data from fishing tournaments held in the United States were obtained from the B.A.S.S. Nation series which is composed of 6 divisions (Western, Central, Southern, Northern, Eastern and Mid-Atlantic) that includes anglers from state chapters and member countries from around the globe. Tournament results used in this manuscript were restricted to the Eastern, Mid-Atlantic, and Southern divisions of B.A.S.S., effectively encompassing the native range of Florida bass (*Micropterus floridanus*), largemouth bass (*M*. *salmoides*), portions of the smallmouth bass distribution (*M*. *dolomieu*), and populations containing *M*. *floridanus* x *salmoides* intergrades. Results were omitted from the Western, Central, and Northern divisions to limit comparisons between native (U.S.) and introduced (southern Africa) populations.

The status of the bass population in each water body was inferred using the number of fish retained per angler per day, a form of catch per unit effort (CPUE), as a proxy for abundance [25]. We used catch records to calculate the combined weight of all fish caught per individual angler per day (average bag weight) and the average fish weight (total weight of fish retained divided by the total number of retained fish) for individual water bodies. Comparisons were made using average values generated for individual lakes representing native and introduced populations in North America and southern Africa, respectively. Catch statistics were tested for normality using a Shapiro-Wilk normality test, and equality of variances were measured using an F-test. Differences in mean values (CPUE, average bag weight, average fish weight) were evaluated using a student’s t-test. Statistical tests were performed using the computer software package R [26], and catch estimates were derived using data pooled across species (*M*. *salmoides*, *M*. *floridanus*, *M salmoides* x *M*. *floridanus* intergrades, and *M*. *dolomieu*).

## Results

### Mapping the distribution of black bass in southern Africa

**Fig 1:**
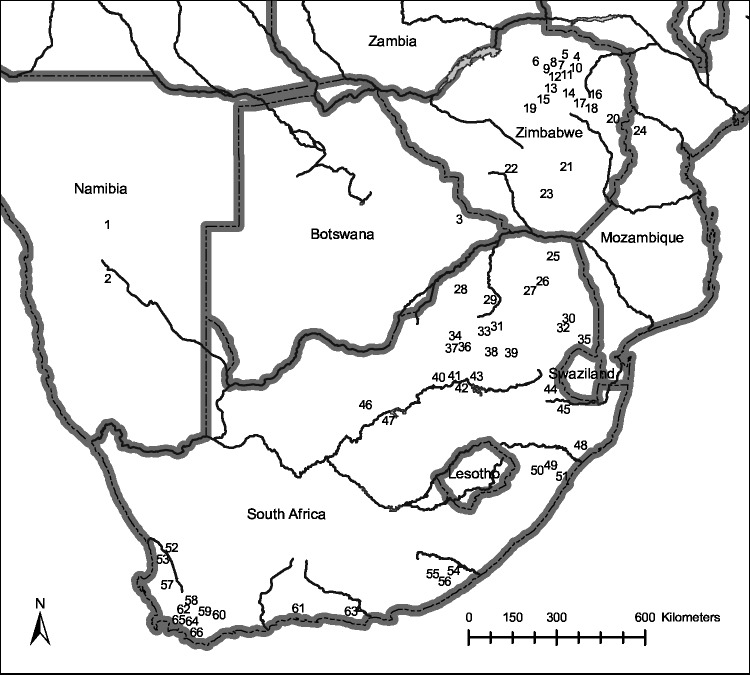
Map of black bass tournament events held in southern Africa. The distribution of black bass (*Micropterus* spp.) angling tournaments held in Botswana, Namibia, Mozambique, South Africa, and Zimbabwe from 1999 to 2013. Location data was projected using ArcGIS v.10.2.2 (ESRI, Redlands, CA, USA), and details (latitude, longitude, water body name) for each point are provided in S1 Table.

Tournament angling events for black bass in southern Africa were documented for 66 impoundments distributed across 5 countries (Fig 1, S1 Table). Data were obtained from 41 impoundments and one lake in South Africa, 20 impoundments in Zimbabwe, two in Namibia, and one each in Botswana and Mozambique. The geographic coordinates of black bass tournaments in southern Africa ranged in latitude from -34.5° to -16.9° South, and in longitude from 16.9° to 33.1° East (Fig 1). In South Africa, tournaments were held in eight of the nine provinces, with the greatest number of tournament venues (i.e. unique impoundments) located in the Western Cape province (12), followed by KwaZulu-Natal (6), Limpopo (5), Mpumalanga (5), North West (5), Eastern Cape (4), Gauteng (4), and Free State (1). Tournaments in Zimbabwe were concentrated in provinces surrounding the capital city of Harare including Mashonaland West (8), Mashonaland Central (5), and Mashonaland East (4). Additionally, tournaments were held in the Masvingo (2) and Matebeleland South (1) provinces of Zimbabwe. Events in Botswana, Namibia, and Mozambique were fewer in number relative to either South Africa or Zimbabwe and displayed no geographic clustering.

### Population success of alien-invasive black bass in southern Africa relative to native populations

Catch records used to compare population metrics were drawn from 40 events hosted by B.A.S.S., S.A.B.A.A., and Z.N.B.F. A total of 41 tournament days were compiled from 15 B.A.S.S. Divisional tournaments in the United States between 2011 and 2014. US records equated to 3,845 days of angler effort. Tournament data from South Africa were collected from a total of 19 water bodies consisting of 97 tournaments days. Additionally, data were compiled from a total of 48 tournament days held on six water bodies in Zimbabwe. Combined, a total of 11,045 days of angler effort were recorded for tournaments held in southern Africa.

**Fig 2:**
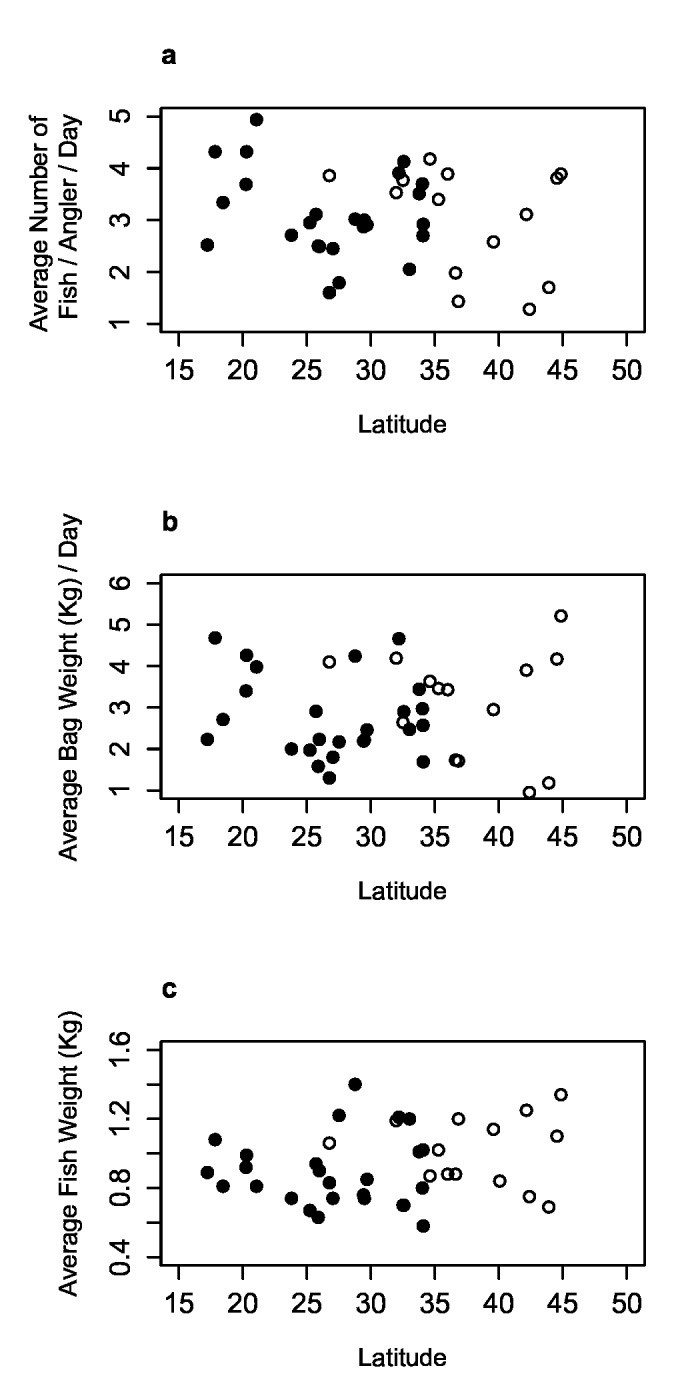
Relative success of alien-invasive and native populations of black bass. Average number of fish retained per angler per day (a), the average daily bag weight of retained fish (b), and the average weight of a fish retained in black bass (*Micropterus* spp.) tournaments held in the United States (white dots) and southern Africa (black dots) (c).

**Fig 3:**
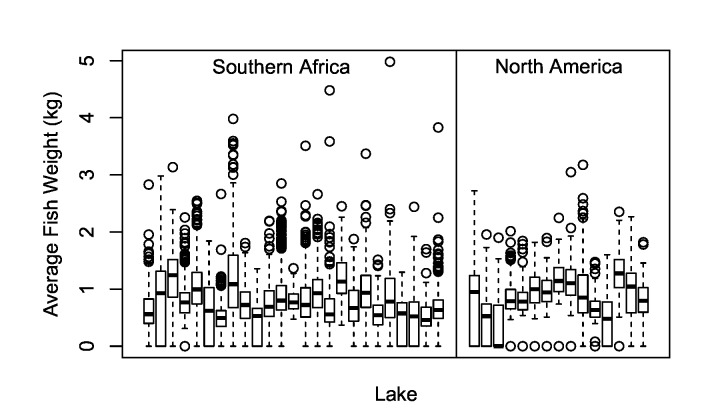
Catch characteristics from tournament angling events held in southern Africa and North America. The average weight of black bass (*Micropterus* spp.) retained in 186 tournament angling events hosted by B.A.S.S. in North America and S.A.B.A.A. and Z.N.B.F. in southern Africa. Each column represents an individual impoundment, and boxes display the 25^th^ and 75^th^ quartiles. Dark lines in boxes indicate median values, whiskers denote the maximum and minimum values excluding outliers. Open circles represent outliers (values more than 1.5 times greater or lower than the maximum or minimum quartiles respectively).

Average values for the number of fish retained per angler per day, daily bag weight, and individual fish weight by lake were comparable between countries (Fig 2, Table 1). On average, an angler participating in a U.S. bass tournament retained 2.87 fish (SD = 1.17) per day with an average daily bag weight of 2.92 total kg of fish per angler (SD = 1.40). For southern Africa, the average number of fish caught per angler per day was 3.10 (SD = 0.82) with an average daily bag weight of 2.76 kg (SD = 0.97). The average individual fish weight was 0.99 kg (SD = 0.21) in the USA and 0.90 kg (SD = 0.20) in southern Africa. Within individual impoundments, the values for average fish weight were highly variable (Fig 3).

**Table 1:** A list of B.A.S.S. affiliated tournament angling venues with the associated catch rates and fish weights from water bodies in the introduced (southern Africa) and native range (United States) of *Micropterus salmoides*, *M*. *floridanus*, and *M*. *dolomieu*.

Region	Water Body	Province, Country	Average Daily Number of Fish Retained	Average Daily Bag Limit Weight (kg)	Average Fish Weight (kg)	Number of angler catch records	Number of Events
Southern Africa							
	Masvikadei	Mashonaland West, ZW	2.52 (1.50)	2.23 (1.93)	0.89	436	4
	Darwendale	Mashonaland West, ZW	4.32 (1.09)	4.68 (2.30)	1.08	1139	10
	Claw	Mashonaland West, ZW	3.34 (1.41)	2.71 (1.53)	0.81	605	6
	Kyle	Masvingo, ZW	3.69 (1.36)	3.40 (2.02)	0.92	1407	18
	Mayfair	Matabeleland South, ZW	4.32 (1.12)	4.26 (2.08)	0.99	912	9
	Manyuchi	Masvingo, ZW	4.94 (0.36)	3.98 (0.99)	0.81	98	1
	Tzaneen	Limpopo, ZA	2.71 (2.30)	2.00 (1.81)	0.74	102	1
	Rust de Winter	Limpopo, ZA	2.95 (2.04)	1.97 (1.55)	0.67	200	2
	Driekoppies	Mpumalanga, ZA	3.11 (2.28)	2.91 (2.69)	0.94	128	1
	Witbank	Mpumalanga, ZA	2.50 (1.86)	1.58 (1.44)	0.63	168	3
	Mokolo	Limpopo, ZA	2.49 (1.84)	2.23 (2.07)	0.90	202	3
	Vaal River	Gauteng, ZA	1.60 (1.78)	1.30 (1.66)	0.83	199	3
	Heyshope	Mpumalanga, ZA	2.45 (2.09)	1.80 (1.93)	0.74	38	1
	Bivane	KwaZulu-Natal, ZA	1.79 (1.69)	2.17 (2.23)	1.22	168	2
	Goedertrouw	KwaZulu-Natal, ZA	3.02 (1.92)	4.24 (3.58)	1.40	358	4
	Albert Falls	KwaZulu-Natal, ZA	2.87 (1.94)	2.19 (1.93)	0.76	364	4
	Midmar	KwaZulu-Natal, ZA	3.00 (1.90)	2.21 (1.87)	0.74	236	3
	Inanda	KwaZulu-Natal, ZA	2.91 (1.92)	2.46 (1.86)	0.85	176	1
	Clanwilliam^a^	Western Cape, ZA	3.91 (1.47)	4.66 (2.49)	1.21	816	11
	Wriggleswade	Eastern Cape, ZA	4.13 (1.47)	2.90 (1.52)	0.70	841	11
	Misverstand	Western Cape, ZA	2.05 (1.19)	2.47 (1.64)	1.20	121	6
	Quaggaskloof^a^	Western Cape, ZA	3.51 (1.59)	3.44 (2.13)	1.01	615	14
	Groenvlei	Western Cape, ZA	3.70 (1.75)	2.97 (1.91)	0.80	685	9
	Theewaterskloof	Western Cape, ZA	2.70 (1.66)	2.57 (2.30)	1.02	280	9
	Elandsjacht	Eastern Cape, ZA	2.92 (1.99)	1.69 (1.30)	0.58	731	9
		Average	3.10 (0.82)	2.76 (0.97)	0.90		
		Total				11,045	145
North America							
	Lake Okeechobee	Florida, US	3.86 (1.76)	4.10 (2.80)	1.06	342	1
	Lake Eufaula	Alabama, US	3.53 (1.62)	4.19 (2.34)	1.19	206	1
	Ouachita River	Louisiana, US	3.77 (1.57)	2.64 (1.45)	0.70	162	1
	Wheeler Lake	Alabama, US	4.18 (1.39)	3.63 (1.85)	0.87	168	1
	Lake Dardanelle	Arkansas, US	3.40 (1.53)	3.46 (1.92)	1.02	168	1
	Douglas Lake	Tennessee, US	3.89 (1.62)	3.43 (1.84)	0.88	328	1
	Kerr Reservoir	Virginia, US	1.98 (1.51)	1.73 (1.42)	0.88	156	1
	Barren River	Kentucky, US	1.43 (1.46)	1.71 (1.86)	1.20	342	1
	Upper Chesapeake River	Maryland, US	2.58 (1.93)	2.95 (2.33)	1.14	294	1
	Delaware River	New Jersey, US	0.60 (0.91)	0.50 (0.78)	0.84	255	1
	Lake Erie^a^	Pennsylvania, US	3.11 (1.39)	3.90 (2.03)	1.25	240	1
	Charles River	Massachusetts, US	1.28 (1.40)	0.95 (1.07)	0.75	358	1
	Sebago Lake	Maine, US	1.70 (1.59)	1.18 (1.43)	0.69	369	1
	Lake Champlain	Vermont, US	3.81 (1.89)	4.17 (2.46)	1.10	226	1
	St. Lawrence River^a^	New York, US	3.89 (1.57)	5.21 (2.60)	1.34	231	1
		Average	2.87 (1.17)	2.92 (1.40)	0.99		
		Total				3,845	15

ZW = Zimbabwe, ZA = South Africa, US = United States.

^a^ indicates a reservoir where the predominant tournament catch is *Micropterus dolomieu*.

Average values and standard deviations (in parenthesis) for number of fish captured and bag limit weights were derived from angler catch records with one record equivalent to one angler day of tournament fishing. Water bodies are listed in latitudinal order within each country such that water bodies high on the list are closest to the equator.

Results from Shapiro-Wilk tests of normality indicated the average number of fish retained per angler (*W* = 0.98, *p* = 0.64,), the average daily bag weight (*W* = 0.98, *p* = 0.68), and average fish weight (*W* = 0.96, *p* = 0.17) were approximately normally distributed. The F-test for equality of variance indicated no significant differences between values for the average number of fish retained per angler (*F* = 0.49, *p* = 0.12, *df* = 24, 14), the average daily bag weight (*F* = 0.48, *p* = 0.11, *df* = 24, 14), and average fish weight (*F* = 0.97, *p* = 0.91, *df* = 24, 14). The mean number of fish retained per tournament angler per day was not significantly different between the United States and southern Africa (*t* = 0.73, *p* = 0.47, *df* = 38). Similarly, no significant difference was observed for the average daily bag weight (*t* = -0.41, *p* = 0.68, *df* = 38) or the average fish weight (*t* = -1.43, *p* = 0.16, *df* = 38).

## Discussion

### Mapping the distribution of black bass in southern Africa

Although important for management applications, alien fishes in impoundments are often underrepresented in formal collections because alien fishes and man-made habitats are often low priorities for ichthyological expeditions. In a collation of southern African databases on fish collections, Scott et al. [27] listed 211 discrete localities for *Micropterus* spp., only 18 (<9%) were from the vicinity of impoundments and none of which were listed in this paper. More centralized, global databases including the United Nations Food and Agriculture Organization’s Database of Invasive Aquatic Species (DIAS), and the Global Invasive Species Database (GISD, http://www.issg.org/database/welcome/) are repositories of information regarding the distribution and transfer of introduced and invasive species worldwide [28,29]. An examination of both the DIAS and GISD records (accessed: November 11^th^ 2014) indicated populations of *Micropterus* spp. are known to occur in Botswana, Namibia, Mozambique, South Africa, and Zimbabwe; however, specific water bodies were not identified and many distribution records were based on dated sources (e.g. [30]). Incorporating location records from tournament angling events may be used to add fine scale resolution to existing records particularly for underrepresented habitats such as impoundments.

Through the novel application of readily available tournament angling catch data, knowledge on the distribution and establishment of centrarchid populations in southern Africa has been expanded to include bass populations from 66 impoundments located in Botswana, Namibia, Mozambique, South Africa, and Zimbabwe. These records not only documented the distribution of invasive species within individual countries but also illustrate the spread of centrarchids across southern Africa, and strongly suggest the possibility of obtaining distribution data worldwide. While the total extent of bass angling tournaments was not evaluated in this paper, annual bass fishing world championships include teams from Russia, Croatia, Germany, France, Spain, Italy, Portugal, Mexico, Venezuela, Swaziland, and South Africa [24]. Given that the selection of teams is based on individual performance at qualifying tournaments, the application of tournament catch data to document the introduction of recreationally important invasive fish species has potential utility at the global scale.

A significant driver in the spread of largemouth bass populations around the globe has been the creation of angling opportunities [15, 22]. In South Africa for example, largemouth bass were transferred extensively for angling purposes through government subsidized stocking programs until the 1980s [14]. Despite the termination of formal stocking programs, populations have since been founded as a result of unauthorized, angler-mediated translocations [14]. Given the informal nature of many population establishments, records of fish transfers are commonly nonexistent, and an alternative means of documenting occurrence and relative abundance is required. Cataloging establishments as they arise is of utmost importance because basic biological data (e.g. population age) form the foundation for research examining the patterns and processes associated with the spread of invasive species [7, 31]. Spatially explicit data as generated from up-to-date tournament results may enhance the ability to track patterns associated invasion pathways [28], identify sources of invaders [32], and project the spread of populations through the use of predictive models (e.g. [33, 34]).

### Population success of alien-invasive black bass in southern Africa relative to native populations

Monitoring programs are regularly used by fisheries research and management agencies to estimate size structure, abundance, and distribution of sport fish species as a means of tracking population trends through time. Relative fish abundance is routinely assessed using metrics of catch per unit effort [35], and when sampling protocols are standardized, data derived from various sampling procedures can be used to draw comparisons of populations across water bodies, seasons, and years [36]. The data used in this paper represents a standardized form of sampling for the following reasons: (1) the governing bodies that regulate bass tournaments (B.A.S.S., S.A.B.A.A., and Z.N.B.F.) enforce rules that are nearly identical; (2) anglers on both continents compete using rods, reels, lures, and boats that are equivalent; and (3) anglers from North America and Africa are of similar skill, as evidenced by the competitive placement by anglers from southern African in North American tournaments (E. Gilliland of B.A.S.S., personal communication, June 16 2014). Combined with relatively large sample sizes in both numbers of tournaments and numbers of fish measured, these attributes indicate than any differences in catch rates were not likely the result of inconsistencies in tournament formats but rather were a reflection of the abundance and size of fish in the populations.

Using bass tournament angling catch data, this analyses demonstrated that catch rates and fish sizes in southern African bass populations were not statistically different from those in native populations in the United States. The range of catch estimates (weights and numbers of fish caught per angler) suggested the relative abundance of largemouth bass in their introduced range were similar to values observed in their native range. Although these data failed to generate values of absolute abundance for the documented populations, the success of invasive black basses in select southern African water bodies has been further documented.

Despite the consistent structure of tournament events, one noted shortfall associated with using tournament data to track population trends is the issue of sampling bias [37]. Researchers have identified that tournament catch data can be used to evaluate largemouth bass size structure and density [37], but that catch sizes and catch rates differ relative to other techniques such as electrofishing or non-tournament angler data [17,38,18]. Despite this shortcoming, it is important to note that such biases would be expected to be similar across all tournaments, and smaller fish are likely to be selected against in tournament catches regardless of water body [39]. As a result, the relative differences in catch rates, daily bag weights, and fish sizes between North American and southern African angling tournaments should be considered readily comparable and useful for population-level comparisons.

### Using tournament records to document bass establishment

Criteria used to assess the status of introduced populations has received considerable attention [6, 40, 41]. A unified framework was proposed by Blackburn et al. [6] used a series of guidelines, including descriptions of stages, barriers, and terminology associated with biological invasions to assess population status. In order for an invasive population to be classified as established there must be evidence of 1) reproduction in the wild, 2) persistence through time, and 3) spread throughout the recipient environment. Using these guidelines, data from black bass tournaments appear to provide sufficient evidence to classify a population as established based on characteristics of the catch data.

Documenting the presence of reproduction in the wild is typically assessed via biological surveys that record the presence of young of year or juvenile fish (e.g. [42]). Tournament angling data from southern Africa impoundments provided intuitive evidence that successful reproduction has occurred. Specifically, average fish weights were distributed over a wide range of values and the heaviest fish (or maximum average fish weight) retained in a given water body was on average several kilograms greater that the lightest fish (or minimum average fish weight) from the same impoundment (Fig 3). Although growth rates of bass are known to vary [43, 44], the spread of average fish weights strongly suggests that multiple age classes were present in the system [45].

Meeting the requirement of population persistence through time may be satisfied using multiple years of tournament data collected from the same water body. Tournament angling data are highly regulated and can be considered analogous to catch per unit effort (CPUE) used in commercial fisheries to track fish stock abundance through time [37]. The use of tournament data to track population trends has been limited [46]; however, records from spearfishing competitions off the coast of Spain were used to document the long-term decline in rocky reef fish abundance and weights [47]. Similarly, Olson and Cunningham [48] used fishing contest results to document declines in catch sizes for a suite of freshwater species in Minnesota lakes over a 58-year period. Angling tournaments that involve greater than 50 participants, have high catch rates, and are held on a regular basis are considered a reliable source of information on population level parameters for largemouth bass [20]. Therefore, the persistence of populations could be illustrated using a standardized form of tournament catch rates (e.g. number of fish/angler/day) recorded over time.

The final criteria for establishment, documenting the spread of individuals in the recipient environment, can be satisfied using the frequency of angling tournaments and the number of anglers participating in these events. Studies examining the spatial distribution of freshwater anglers have typically operated over broad geographic scales (e.g. [49, 50]), and examination of angler distributions within impoundments are few (e.g. [51]). Taylor et al. [24] showed the spatial distribution of angling effort by tournament anglers in a South African lake was scattered throughout the water body. These results indicated fish captures in tournament fishing were likely derived from multiple areas within a given impoundment. Water bodies where black bass tournaments are held typically host events at regular intervals (i.e. multiple times per year) and can draw large numbers of participants (e.g. >100 anglers) [52]. Taking the number of events and anglers combined, one may reasonably assume that fish are caught all over a given water body, indicating bass have spread throughout the recipient environment.

Despite the utility of tournament angling data in characterizing the status of invasive sport fish populations, several limitations associated with the data warrant discussion. First, angling tournaments are generally focused on water bodies with established populations, most of which are restricted to impoundments and not rivers. Interestingly, many of the tournaments held in the native US range were held on river systems (6 of 15), however events from southern Africa were almost entirely restricted to impoundments. That tournaments are focused on established populations and not those recently introduced implies tournament data is inappropriate to detect populations early in the invasion process [6]. Nonetheless, cataloging the distribution of invasive populations is necessary for the development of predictive models of spread which can serve to direct prevention, monitoring, and quarantine efforts [33]. Second, tournaments described in this manuscript were solely focused on black basses (*Micropterus* spp.); however, methodologies presented here can be readily applied to other invasive sport fish species targeted by competitive angling events. Lastly, many of the water bodies targeted by tournaments must be adequate in size to accommodate a moderate to large number of anglers. However, by incorporating data from local, regional, and national level events, data were drawn from events which spanned a range of sizes (both in water body size and number of angler participants). In spite of the above mentioned shortfalls, tournament angling data may serve to enhance our understanding of the distribution and population characteristics of invasive sport fishes worldwide.

The use of tournament data to assess invasion status represents a novel and cost effective method to document trends in fish populations in non-native waters relative to their home range. Given the global distribution of invasive black bass and widespread popularity of competitive angling, catch data derived from tournaments may serve as a valuable tool to update distribution records, assess invasions status, and track population trends through time. Generating real-time data from tournament catch data may aid in documenting illegal or unauthorized fish transfers, especially in areas where fisheries agency funds and personnel are limited. Future studies of invasion ecology should consider utilizing existing angler catch data for documenting the spatial and temporal trends in fish invasions.

## Supporting Information

S1 Table Details associated with water bodies hosting tournament angling events in southern Africa. A list of water bodies that hosted local club, regional, or national black bass (*Micropterus* spp.) tournament angling events between 1999 and 2013 in southern Africa and their associated GPS coordinates. Population number refers to water bodies identified in Fig 1.
(DOCX)
