# Supplementary material for: Using Tournament Angler Data to Rapidly Assess the Invasion Status of Alien Sport Fishes (Micropterus spp.) in Southern Africa
Source: PLoS One. 2015 Jun 5;10(6):e0130056. doi: 10.1371/journal.pone.0130056 (PMC4457859; doi:10.1371/journal.pone.0130056)
Supplement: S1 Table — A list of water bodies that hosted local club, regional, or national black bass (Micropterus spp.) tournament angling events between 1999 and 2013 in southern Africa and their associated GPS coordinates. Population number refers to water bodies identified in Fig 1. (DOCX) [file pone.0130056.s001.docx]

| Water Body | Province, Country | Latitude | Longitude | Population Number |
| --- | --- | --- | --- | --- |
| Von Bach | Otjozondjupa Region, NA | -22.013900 | 16.953300 | 1 |
| Oanab | Hardap Region, NA | -23.325462 | 17.023218 | 2 |
| Letsibogo | Central Region, BW | -21.845184 | 27.719744 | 3 |
| Machere | Mashonaland Central, ZW | -16.911326 | 31.216403 | 4 |
| Arcadia | Mashonaland Central, ZW | -17.006250 | 31.012917 | 5 |
| Two Tree | Mashonaland West, ZW | -17.007222 | 30.059444 | 6 |
| Masembura | Mashonaland Central, ZW | -17.108750 | 31.021250 | 7 |
| Suji | Mashonaland West, ZW | -17.187509 | 30.454445 | 8 |
| Masvikadei | Mashonaland West, ZW | -17.220556 | 30.386667 | 9 |
| Mwenje | Mashonaland Central, ZW | -17.260000 | 31.033611 | 10 |
| Kia-Ora | Mashonaland Central, ZW | -17.338731 | 30.989536 | 11 |
| Bassinwood | Mashonaland West, ZW | -17.420333 | 30.661889 | 12 |
| Darwendale | Mashonaland West, ZW | -17.827425 | 30.521253 | 13 |
| Henry Hallam | Mashonaland West, ZW | -17.984675 | 31.067479 | 14 |
| Poole | Mashonaland West, ZW | -18.170656 | 30.283264 | 15 |
| Winnembi | Mashonaland East, ZW | -18.263314 | 31.648911 | 16 |
| Dudley | Mashonaland East, ZW | -18.317675 | 31.468800 | 17 |
| Sable Park | Mashonaland East, ZW | -18.440742 | 31.596706 | 18 |
| Claw | Mashonaland West, ZW | -18.446571 | 29.868611 | 19 |
| Osborne | Mashonaland East, ZW | -18.766389 | 32.485556 | 20 |
| Kyle | Masvingo, ZW | -20.237309 | 31.014331 | 21 |
| Mayfair | Matabeleland South, ZW | -20.331667 | 29.255000 | 22 |
| Manyuchi | Masvingo, ZW | -21.062525 | 30.392385 | 23 |
| Chicamba Real | Manica, MZ | -19.130437 | 33.107031 | 24 |
| Nandoni | Limpopo, ZA | -22.981033 | 30.598064 | 25 |
| Tzaneen | Limpopo, ZA | -23.800000 | 30.163610 | 26 |
| Ebenezer | Western Cape, ZA | -23.940409 | 29.977667 | 27 |
| Mokolo | Limpopo, ZA | -23.982747 | 27.756395 | 28 |
| Doorndraai | Limpopo, ZA | -24.299806 | 28.756928 | 29 |
| Injaka | KwaZulu-Natal, ZA | -24.884505 | 31.072206 | 30 |
| Renosterkop | Mpumalanga, ZA | -25.109836 | 28.887350 | 31 |
| Da Gama | Mpumalanga, ZA | -25.141977 | 31.017742 | 32 |
| Rust de Winter | Limpopo, ZA | -25.237182 | 28.514130 | 33 |
| Roodekoppies | North West, ZA | -25.410776 | 27.590511 | 34 |
| Driekoppies | Mpumalanga, ZA | -25.716667 | 31.522156 | 35 |
| Hartbeespoort | North West, ZA | -25.747732 | 27.887178 | 36 |
| Buffelspoort | North West, ZA | -25.791352 | 27.489123 | 37 |
| Bronkhorstspruit | Gauteng, ZA | -25.897426 | 28.695474 | 38 |
| Witbank | Mpumalanga, ZA | -25.922464 | 29.310514 | 39 |
| Potchefstroom | North West, ZA | -26.670000 | 27.097222 | 40 |
| Vaal Barrage | Gauteng, ZA | -26.764722 | 27.691667 | 41 |
| Vaal River | Gauteng, ZA | -26.764722 | 27.684117 | 42 |
| Vaal Dam | Gauteng, ZA | -26.888990 | 28.121382 | 43 |
| Heyshope | Mpumalanga, ZA | -27.039591 | 30.517215 | 44 |
| Bivane | KwaZulu-Natal, ZA | -27.519284 | 31.053518 | 45 |
| Taung | North West, ZA | -27.520261 | 24.851589 | 46 |
| Bloemhoek | Free State, ZA | -27.696904 | 25.687065 | 47 |
| Goedertrouw | KwaZulu-Natal, ZA | -28.764444 | 31.429444 | 48 |
| Albert Falls | KwaZulu-Natal, ZA | -29.445449 | 30.426940 | 49 |
| Midmar | KwaZulu-Natal, ZA | -29.500000 | 30.183333 | 50 |
| Inanda | KwaZulu-Natal, ZA | -29.706080 | 30.873070 | 51 |
| Bulshoek | Western Cape, ZA | -32.037590 | 18.818311 | 52 |
| Clanwilliam | Western Cape, ZA | -32.197222 | 18.879691 | 53 |
| Wriggleswade | Eastern Cape, ZA | -32.594022 | 27.552520 | 54 |
| Binfield | Eastern Cape, ZA | -32.691703 | 26.907186 | 55 |
| Rooikrantz | Eastern Cape, ZA | -32.752948 | 27.325853 | 56 |
| Misverstand | Western Cape, ZA | -33.025649 | 18.788926 | 57 |
| Quaggaskloof | Western Cape, ZA | -33.779167 | 19.432879 | 58 |
| Breede | Western Cape, ZA | -33.844988 | 19.934993 | 59 |
| Poortjies | Western Cape, ZA | -33.866638 | 20.372086 | 60 |
| Groenvlei | Western Cape, ZA | -34.030389 | 22.851942 | 61 |
| Theewaterskloof | Western Cape, ZA | -34.033580 | 19.253710 | 62 |
| Elandsjacht | Eastern Cape, ZA | -34.062469 | 24.641558 | 63 |
| Whitehall | Western Cape, ZA | -34.241963 | 19.094925 | 64 |
| Provost | Western Cape, ZA | -34.244162 | 19.076900 | 65 |
| Kraaibos | Western Cape, ZA | -34.563404 | 19.493952 | 66 |

Country codes are as follows: BW = Botswana, NA = Namibia, MZ = Mozambique, ZA = South Africa, ZW = Zimbabwe. Population number refers to location labels found in Fig. 1.
